# A cell-body groove housing the new flagellum tip suggests an adaptation of cellular morphogenesis for parasitism in the bloodstream form of *Trypanosoma brucei*

**DOI:** 10.1242/jcs.139139

**Published:** 2013-12-15

**Authors:** Louise Hughes, Katie Towers, Tobias Starborg, Keith Gull, Sue Vaughan

**Affiliations:** 1 Department of Biological and Medical Sciences, Faculty of Health and Life Science, Oxford Brookes University, Oxford OX3 0BP, UK; 2 Wellcome Centre for Cell Matrix Research, Michael Smith Building, Manchester M13 9PT, UK; 3 Sir William Dunn School of Pathology, University of Oxford, South Parks Road, Oxford OX1 3RE, UK

**Keywords:** *Trypanosoma brucei*, Flagellum, Axoneme, Serial block face scanning electron microscopy, SBF-SEM, Microtubule

## Abstract

Flagella are highly conserved organelles present in a wide variety of species. In *Trypanosoma brucei* the single flagellum is necessary for morphogenesis, cell motility and pathogenesis, and is attached along the cell body. A new flagellum is formed alongside the old during the cell division cycle. In the (insect) procyclic form, the flagella connector (FC) attaches the tip of the new flagellum to the side of the old flagellum, ensuring faithful replication of cell architecture. The FC is not present in the bloodstream form of the parasite. We show here, using new imaging techniques including serial block-face scanning electron microscopy (SBF-SEM), that the distal tip of the new flagellum in the bloodstream form is embedded within an invagination in the cell body plasma membrane, named the groove. We suggest that the groove has a similar function to the flagella connector. The groove is a mobile junction located alongside the microtubule quartet (MtQ) and occurred within a gap in the subpellicular microtubule corset, causing significant modification of microtubules during elongation of the new flagellum. It appears likely that this novel form of morphogenetic structure has evolved to withstand the hostile immune response in the mammalian blood.

## Introduction

Cilia and flagella are highly conserved eukaryotic organelles that generate movement, extracellular flow and cell signaling events for a wide variety of cell types. They are crucial for normal human development, and defects in their assembly and function have been linked to a number of human diseases, collectively known as ciliopathies (for a review, see Waters and Beales, 2011). Flagella are also important for the pathogenicity of a number of eukaryotic parasites. In these parasites flagella have sensory and motility functions important for cell–cell recognition, pathogenicity, host–vector transfer mating, and invasion of different tissue environments in host and vector. *Trypanosoma brucei* is a protozoan parasite that causes a devastating human and animal disease in sub-Saharan Africa. *T. brucei* has a single motile flagellum that is required for cell motility, enabling the cell to move within the vertebrate host and insect vector. The flagellum and associated flagellar pocket are key virulence factors involved in host–parasite interactions, and are implicated in immune evasion mechanisms in mammalian blood. It is also important for cellular morphogenesis and propagation of the parasite (Broadhead et al., 2006; Engstler et al., 2007; Moreira-Leite et al., 2001; Ralston et al., 2009; Sharma et al., 2008; Sherwin and Gull, 1989; Vaughan et al., 2008; Vickerman, 1969b).

The flagellum of the two trypomastigote forms of the parasite – the procyclic (insect) form and mammalian bloodstream form contains a 9+2 microtubule axoneme that is highly conserved across eukaryotic species. It also contains an additional lattice-like structure called the paraflagellar rod (PFR), which is attached to microtubule axoneme doublets four through seven (Hughes et al., 2012; Portman et al., 2009; Vickerman, 1969b). The flagellum exits the cell body through an invagination of the plasma membrane called the flagellar pocket (Field and Carrington, 2009; Lacomble et al., 2009). Whereas most eukaryotic flagella extend out from a cell into the surrounding medium, the flagellum of *T. brucei* exits the flagellar pocket and is attached along the long axis of the cell body by the flagellum attachment zone (FAZ) (Taylor and Godfrey, 1969). This encompasses a FAZ filament and a set of four specialized microtubules called the microtubule quartet (MtQ). In the flagellum, filamentous structures link the flagellar membrane to the PFR (Kohl et al., 1999). Between the flagellar and cell body membranes, extracellular structures described as ‘staples’ are present along the entire length of the attachment zone in the insect (procyclic) form (Höög et al., 2012).

On the cytoplasmic side of the attachment zone in both life cycle forms there are dense macular structures and filaments associated with the cell body membrane. To the left of the FAZ filament (when the cell is viewed from the posterior end of the cell) is the set of four specialized microtubules (Taylor and Godfrey, 1969; Vickerman, 1969b), now known as the microtubule quartet (MtQ) (Lacomble et al., 2009). These originate between the basal bodies at the proximal end of the flagellum, extend around the flagellar pocket and insert into the subpellicular corset of microtubules (Lacomble et al., 2009; Taylor and Godfrey, 1969; Vickerman, 1969b). The corset microtubules, organized in a planar array that lies beneath the plasma membrane, are cross-linked to each other and the plasma membrane with their plus ends positioned facing the posterior end of the cell (Robinson et al., 1995; Sherwin and Gull, 1989; Vickerman, 1969a). The microtubules in the MtQ are anti-parallel to the corset microtubules, with their plus ends at the anterior end of the cell (Robinson et al., 1995), and are morphologically identified by the close association of a sheet of endoplasmic reticulum, which interdigitates between each of the four microtubules (Lacomble et al., 2009; Taylor and Godfrey, 1969; Vickerman, 1969b). The combination of the FAZ filament and MtQ creates a distinct seam within the subpellicular microtubule array that runs from the flagellar pocket to the anterior end of the cell.

The cytoarchitecture of *T. brucei* remains largely intact during the cell division cycle. This necessitates a highly ordered sequence of duplication and segregation events of the mainly single copy organelles and cytoskeleton to form two daughter cells from within the confines of the existing cell. In *T. brucei*, growth of the new flagellum and attachment to the cell body has emerged as a key factor in cell morphogenesis. The new flagellum is always located to the left of the old flagellum when the cell is viewed from the posterior end and follows a left-handed helix up the cell (Sherwin and Gull, 1989; Vickerman, 1969a). The mechanism behind the strict cytological arrangement of the new flagellum is beginning to be understood with the discovery of a mobile transmembrane junction called the flagella connector in the procyclic (insect form) cells (Moreira-Leite et al., 2001). As the new flagellum grows, the distal tip is attached to the old flagellum through the flagella connector. The physical attachment of the new flagellum to the old through the flagella connector provides a cytotactic mechanism (Beisson, 2008; Beisson and Sonneborn, 1965; Sonneborn, 1964) that ensures faithful inheritance of the cellular form and facilitates division (Briggs et al., 2004; Moreira-Leite et al., 2001). There are subtle differences in the organization of division between the bloodstream form and procyclic form trypanosome. The flagella connector has not been detected in the bloodstream form and yet the same highly structured inheritance pattern seems to apply to this mammalian proliferative form as it does in the proliferative tsetse midget, procyclic parasite. Given that an understanding of the cell division mechanism will be crucial to understand pathogenicity we decided to examine more closely the possibility of a flagella connector existing in the bloodstream form parasite.

We used a combination of high-resolution conventional transmission electron microscopy (TEM), scanning electron microscopy (SEM), electron tomography and serial block-face SEM (SBF-SEM) (Denk and Horstmann, 2004) to investigate the distal tip of the new flagellum in the mammalian bloodstream form. We found no evidence for a flagella connector analogous to that found in the procyclic form parasite. Rather, we describe the discovery of a discrete invagination of the cell body plasma membrane containing the distal tip of the growing new flagellum, which we have named the groove. The groove was found to be closely associated with MtQ of the FAZ during growth of the new flagellum. We speculate that the groove has a similar function to the flagella connector in providing morphogenetic patterning to the bloodstream form of the parasite, yet is specifically adapted in order to reduce exposure of transmembrane junctions to the immune system. Thus, the use of a groove in bloodstream proliferating trypomastigotes might represent a specific modification of the trypanosome division process for the pathogenicity in the mammalian bloodstream.

## Results

### The growing new flagellum distal tip is located in a distinct groove enclosed by the cell body membrane

**Fig. 1.**
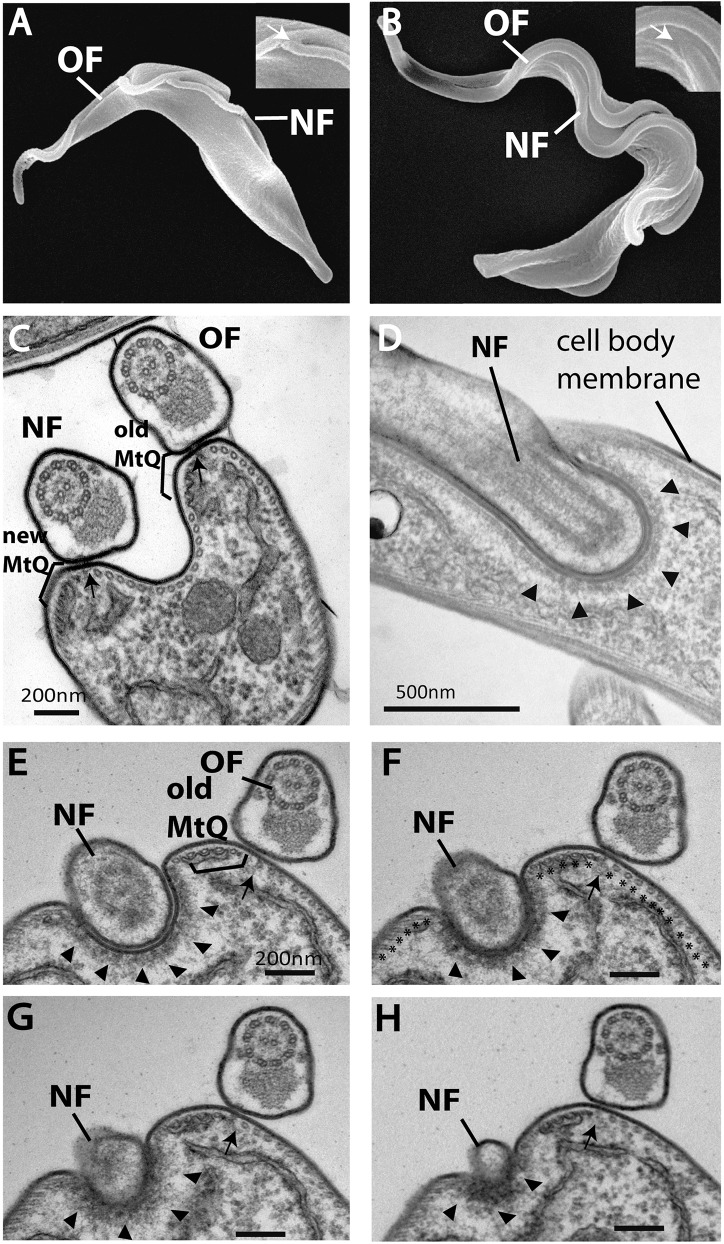
**The distal tip of the new flagellum in bloodstream form cells is located in an indentation of the cell body membrane.** (A) A SEM image of the procyclic form with an old (OF) and new flagellum (NF). The distal tip of the new flagellum is attached to the old flagellum (arrow in inset). (B) A SEM image of the bloodstream form illustrating the distal tip of the new flagellum lying alongside the old flagellum (arrow in inset). (C) A TEM image showing a cross section through an old and new flagellum illustrating the organization of flagella attachment to the cell body through a FAZ filament (arrow) and MtQ (brackets). (D) Longitudinal TEM thin-section through the distal tip of a new flagellum, illustrating the tip embedded within the cell body (arrowheads). (E–H) Adjacent serial cross sections (∼100 nm thick) through an old flagellum and the distal tip of a new flagellum, illustrating the distal tip located in an indentation within the cell body (arrowheads). The old MtQ (bracket) and old FAZ filament (arrow) are indicated in E. Microtubules are shown by asterisks in F.

The distal tip of the growing new flagellum of the insect (procyclic) form of the parasite is connected to the old flagellum through a mobile transmembrane connection called the flagella connector (Fig. 1A, arrow in inset) (Briggs et al., 2004; Moreira-Leite et al., 2001). It has been recognized for some time that the new flagellum of the bloodstream form of the parasite does not connect to the old flagellum (Briggs et al., 2004). However, our analysis of SEM images of dividing trypanosomes (Fig. 1B, arrow in inset) revealed a similar tracking of the new flagellum alongside the old flagellum as it extended. The ultrastructure of the distal tip of the bloodstream form new flagellum was investigated by conventional TEM.

In *T. brucei* a specific orientation allows one to distinguish between the new and old flagellum of dividing cells in TEM cross sections such that when viewed from the posterior end of the cell the new flagellum is always on the left-hand side (Fig. 1C) (Sherwin and Gull, 1989). Cross sections through cells with old and new flagella illustrate a well-characterized cellular architecture in the region of flagellum attachment. Each flagellum has a FAZ filament (Fig. 1C, arrows) that is positioned in a gap in the planar array of cross-linked, regularly spaced subpellicular microtubules (Fig. 1C) and a MtQ is positioned to the left of the FAZ filament of each flagellum (Fig. 1C). Serial cross sections of dividing cells through the distal end of a new flagellum revealed that the tip was located in a discrete indentation of the cell body plasma membrane in the bloodstream form (Fig. 1D–H, arrowheads). The cell body indentation did not contain a FAZ filament connection or subpellicular microtubules; however, microtubules were identified on either side of the indentation (marked with asterisks, Fig. 1F). An electron-dense area was observed underlying the cell body membrane within the indentation. We have named this whole indentation complex the ‘groove’ (Fig. 1D–H, arrowheads). We observed variations in the depth of the groove, for example, in Fig. 1D the tip is embedded in the cell body and in Fig. 1H the tip is in a more shallow indentation. This variation is discussed in more detail later.

### The groove defines a remodeling point in the microtubule corset

**Fig. 2.**
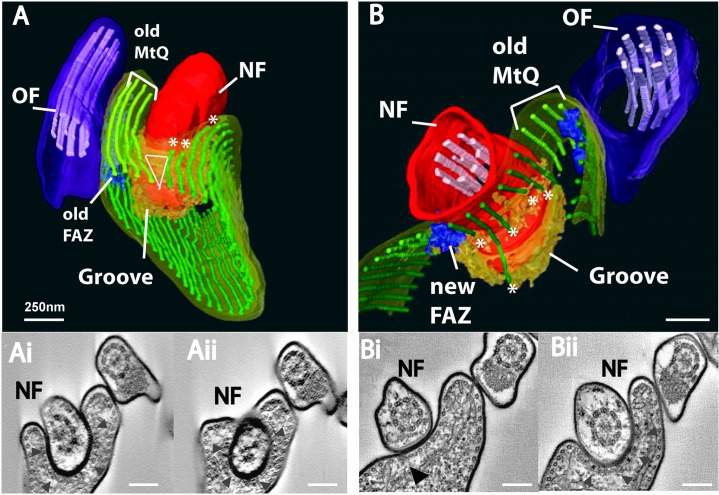
**The subpellicular microtubule cytoskeleton is remodeled during new flagellum growth.** (A,B) Surface renderings of segmented data and selected tomogram slices of two serial section tomograms illustrate the remodeling of subellicular microtubules that surround the groove and the close association of the old MtQ with the groove. (A) Remodeling of subpellicular microtubles anterior to the groove. The subpellicular microtubule array (green), old FAZ (blue), old MtQ (light green), new flagellum (NF) and old flagellum (OF) are shown. A punctuate electron density underlies the cytoplasmic face of the groove (orange). Asterisks mark the subpellicular microtubules that terminate within the tomogram. The triangle illustrates modification of spacing between subpellicular microtubules anterior to the groove. (Ai, Aii) *z*-slices from the tomogram that was used to produce the model shown in A, each slice is ∼6 nm thick. The groove is highlighted (arrowheads). (B) Remodeling of subpellicular microtubules posterior to the groove illustrating terminating subpellicular microtubules (asterisks) and the new FAZ posterior to the groove. The groove is located immediately to the right of the old MtQ. (Bi, Bii) *z*-slices from the tomogram that was used to produce the model shown in A; each slice is ∼6 nm thick. The FAZ associated with the new flagellum and the groove are indicated on the tomogram data. Scale bars: ∼250 nm.

The distal tip of the bloodstream form new flagellum was investigated by cellular electron tomography. Surface rendering of segmented data from serial tomograms (containing three to five adjacent serial 200-nm sections, encompassing 0.6–1 µm) illustrated the major cytoskeletal features both posterior to and surrounding the area of the groove (Fig. 2).

The distal tip of the new flagellum was fully enclosed by the cell body plasma membrane as shown in individual slices from tomograms (arrowheads in Fig. 2Ai,ii) and surface renderings of segmented data (Fig. 2A). The groove was located in a gap in the subpellicular microtubule array, representing a significant modification of the microtubule array as the new flagellum extended along the long axis of the cell (Fig. 2A). Subpellicular microtubules terminated immediately anterior to the groove (Fig. 2A, asterisks), in the direction of new flagellum growth, and posterior to the groove (Fig. 2B, asterisks), where the tip of the new flagellum becomes enclosed by the cell membrane. The emerging gap and modification in spacing between the microtubules anterior to the groove created a triangle of ‘free’ space. This is most clearly illustrated by viewing supplementary material Movie 1. Additionally, Fig. 2B shows that as the microtubules end, the spacing of the posterior subpellicular microtubules becomes wider (Fig. 2B, asterisks; supplementary material Movie 2). No deformation of microtubule spacing was observed between microtubules on either side of the groove, which maintained their regular spatial distribution. No new FAZ filament was associated with the distal tip of the new flagellum (Fig. 2A,B).

**Fig. 3.**
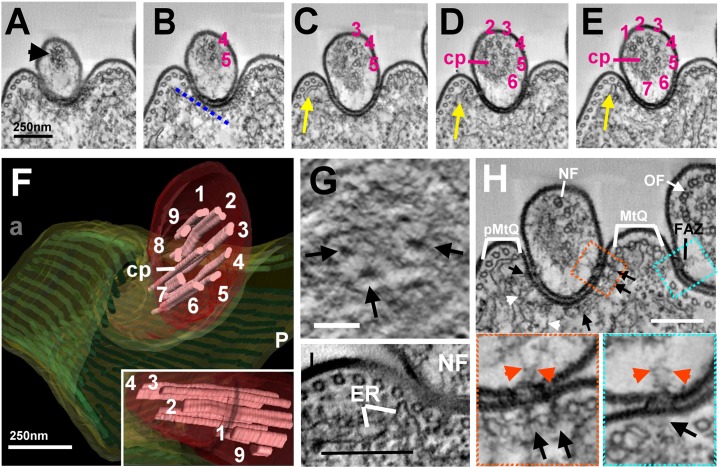
**Organization of the axoneme and ultrastructural organization of the cytoplasmic face of the groove.** (A–E) Slices through a reconstructed tomogram reveal the order of axonemal microtubule assembly. (A) Most distal slice through the new flagellum shows diffuse material (black arrowhead) and no discernible microtubules. (B) The first microtubule doublets to form are four and five. (C–E) Subsequent slices reveal that the other microtubule doublets form in a sequential manner in both a clockwise and anticlockwise direction. Yellow arrows indicate the ER associated with microtubules adjacent to the groove. (D) The central pair (cp) form at the midpoint of the axoneme assembly. (F) A surface volume rendering of the segmented data illustrating the three-dimensional organization at the distal tip; the insert shows a different orientation of the same data. (G) Slice through a tomogram illustrating the punctate maculae on the cytoplasmic face of the groove. The relative position of this slice is indicated by the dashed blue line on B. (H) Cross section of an old and new flagellum. The old FAZ filament (blue boxed inset) and filaments connecting with the cytoplasmic face in the groove area (orange boxed inset) are highlighted (arrows). Corresponding filaments on the flagellum side are indicated with orange arrows. Scale bars: ∼250 nm.

The groove was located adjacent to the MtQ and the FAZ filament associated with the old flagellum (Fig. 2A,B). The FAZ filament associated with the new flagellum terminated posterior to the groove (Fig. 2B). The MtQ are morphologically identified by the close association of a sheet of endoplasmic reticulum (ER), which partially interdigitates between each of the four microtubules (e.g. Fig. 1E) (Taylor and Godfrey, 1969; Vickerman, 1969b). Positive identification of a new MtQ was made difficult by a lack of closely associated ER surrounding each of the four microtubules immediately to the left of the groove in all tomograms (Fig. 3C–E, arrows; 3I). The PFR did not extend to the distal tip of the new flagellum, as shown in tomograms that encompass the groove (Fig. 2A,B). Subpellicular microtubules (green) underlie the cell body plasma membrane throughout this area, with the exception of the line of the FAZ filament (blue).

### An asymmetric organization of axonemal doublets at the distal end of the growing new flagellum

Cellular electron tomography revealed a distinctive asymmetric organization of outer doublet microtubules of the axoneme at the growing new flagellum tip, such that one half of the axoneme extended beyond the other (*n* = 7 tomograms and 18 SBF-SEM datasets) (Fig. 2A; Fig. 3A–F; supplementary material Movie 3). Owing to the invariant linkage of the PFR with the specific outer doublets four through seven (Sherwin and Gull, 1989), and the lack of central pair rotation (Branche et al., 2006; Gadelha et al., 2006; Ralston et al., 2006), we were able to unambiguously assign doublet numbers. At the very distal tip there was a diffuse electron density (Fig. 3A, arrowhead). The most distal microtubule doublets of the axoneme that could be identified were outer doublets four and five (Fig. 3B), then moving through the tomographic reconstructions in a proximal direction away from the distal tip the next longest doublet was doublet three (Fig. 3C) followed by doublets two and six (Fig. 3D). The central pair microtubules appeared approximately at the midpoint of this asymmetric region (Fig. 3D). The shortest doublets were eight and nine (Fig. 3F).

### The cytoplasmic face of the groove plasma membrane exhibits a meshwork of filaments and punctuate structures

Despite the lack of the new FAZ filament in the region of the groove (Figs 1, 2), the plasma membrane of the new flagellum was always closely associated with the cell body plasma membrane, suggesting that there is a mechanism for flagellum attachment within the groove. Using conventional thin-section TEM, an electron-dense area was observed on the cytoplasmic face of the plasma membrane within the groove (Fig. 1D–H). Cellular electron tomography revealed this to possess both punctuate and filamentous electron-dense material (Fig. 3G,H). A slice taken from a tomogram illustrates three of these punctuate electron densities (Fig. 3G, arrows). Filaments radiated out from each density forming an interconnected meshwork covering the entire region of the groove (Fig. 3G). Cross section tomographic slices through the new flagellum within the groove area also demonstrated further filaments connecting with the cytoplasmic face of the cell body plasma membrane (Fig. 3H, orange boxed area, arrows). Similar FAZ filaments exist on the old flagellum area (Fig. 3H, blue boxed area, arrow). Corresponding filaments were also observed on the flagellum side where the two plasma membranes were closely apposed (Fig. 3H, orange and blue boxes, arrowheads).

### The bloodstream form groove is detected by the monoclonal antibody DOT1

**Fig. 4.**
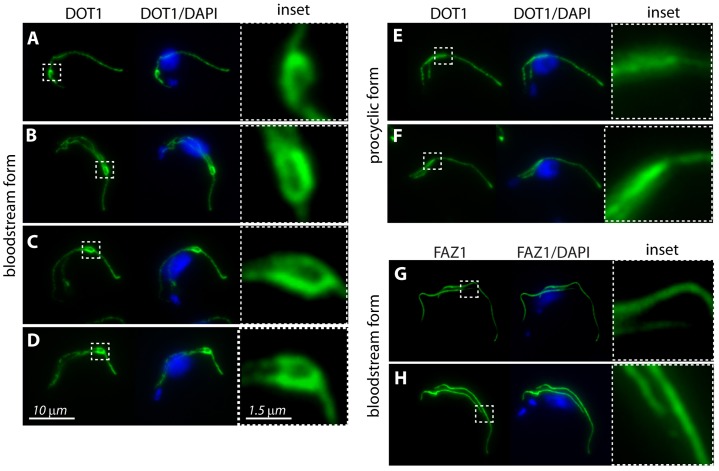
**Immunolabeling reveals an elaboration in DOT1 labeling at the tip of the growing new flagellum in cells of the bloodstream form.** (A–D) DOT1 displays a distinctive curve-shaped elaboration at the distal end of the new flagellum in the bloodstream form cytoskeletons. (E,F) DOT1 labeling in procyclic form cytoskeletons does not display this elaboration at the distal tip of the new flagellum. (G,H) FAZ1 labeling in bloodstream form shows no distinctive elaboration at the distal tip of the new flagellum during its elongation.

ructural similarities of the densities in micrographs of the cytoplasmic face of the groove, and similar repeat densities associated with the FAZ filament, we investigated whether anti-FAZ antibodies labeled the groove. Both procyclic (insect) form and bloodstream form trypanosomes were labeled with the anti-FAZ filament antibody DOT1, which labels the punctuate maculae of the FAZ (Woods et al., 1989), and L3B2, which recognizes the FAZ1 protein (Kohl et al., 1999; Vaughan et al., 2008). We discovered a conspicuous difference in the pattern of labeling using DOT1 antibodies between the life cycle forms. A distinctive curve-shaped elaboration of labeling was observed at the distal end of the bloodstream new flagellum (Fig. 4A–D, inset), whereas the procyclic form exhibited a more linear pattern of labeling (Fig. 4E,F). Identical labeling was observed in both the bloodstream (Fig. 4G,H) and procyclic forms (data not shown) using monoclonal antibodies to the FAZ1 protein.

### The new flagellum tip association is modulated through the cell division cycle

**Fig. 5.**
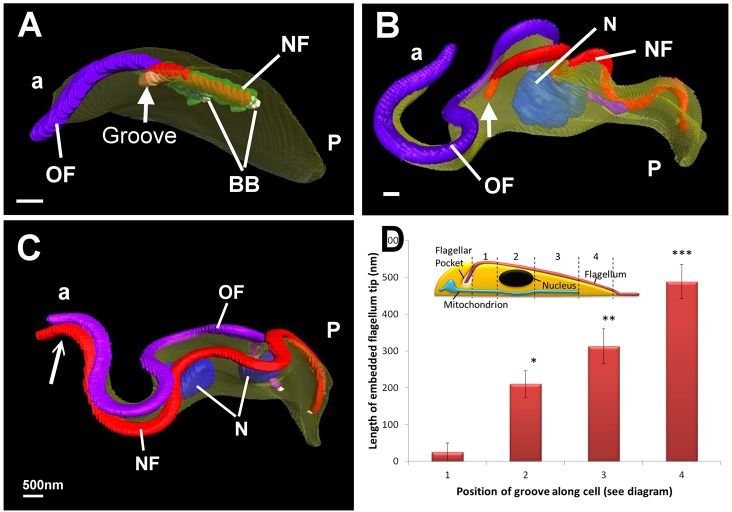
**Morphogenetic characteristics of the groove revealed by SBF-SEM.** (A) Surface volume rendering of SBF-SEM data shows the posterior (P) end of a cell containing the old flagellum (OF, purple) and a short new flagellum (NF, red), emerging from the flagellar pocket (green), basal bodies are also indicated (BB). The new flagellum has its distal tip embedded in a groove (arrow) of plasma membrane (yellow). The anterior end (a) of the section of cell shown is indicated. (B) Surface volume of segmented whole cell. The new flagellum is located in a groove within the body of the cell (arrow). Nuclei (N) are indicated. (C) Cell with a long new flagellum that has extended beyond the anterior tip of the cell (arrow) and lacks evidence of a groove. (D) Chart showing the relative positions (means±s.d.) of the new flagellum tip (1–4 in the diagram and along the *x*-axis of the graph) during flagellum elongation. A statistically significant increase (*, **, *** *P*<0.001) in the length of flagellar tip completely surrounded by the cell body plasma membrane is observed with respect to flagellum length (*n*≥4 for each position). Scale bars: ∼500 nm.

Morphogenetic characteristics of the groove were investigated using SBF-SEM. The technique provides 3D data from large numbers of whole cells at slightly lower resolution than tomography. Volumes containing adjacent serial sections (100-nm thick) of individual trypanosomes with a new flagellum of different lengths were extracted from SBF-SEM datasets and modeled. The morphology was comparable to that obtained by conventional SEM (compare Fig. 5A–C and Fig. 1A,B) and also revealed the internal ultrastructure of the cells. This technique permitted unequivocal identification of the distal tip of the new flagellum and full three-dimensional analysis of the cells and the groove. Analysis of cells with a very short new flagellum that had just exited the flagellar pocket demonstrated that the groove was first located at the point where the distal tip of the new flagellum exited the flagellar pocket. This is best illustrated by viewing supplementary material Movie 4 of the cell shown in Fig. 5A. A groove was observed in all whole-cell reconstructions where the cell possessed a new flagellum that had exited the flagellar pocket, but had not reached the anterior end of the cell. This is best illustrated by viewing supplementary material Movie 5 of the cell imaged in Fig. 5B. In cells where the new flagellum had extended to the anterior end of the cell but had not extended beyond it, a groove structure was evident. However, in cells where the new flagellum was beyond the anterior tip of the cell (Fig. 5C) or had begun cytokinesis no groove was observed.

As indicated above, our data revealed variation in the association of the flagellar tip and the groove. In some cells, the very distal tip of the new flagellum was situated in a shallow indentation (Fig. 1E–H; Fig. 3A–E; supplementary material Movie 3), whereas in other examples the very distal tip was completely embedded within the cell body (Fig. 1D; Fig. 2A; Fig. 5B; supplementary material Movies 1, 5). A comparison of whole trypanosomes from the SBF-SEM datasets revealed that the variation was linked to the length of the new flagellum. We measured the length of the embedded portion (from the point where the flagellum entered the groove to its distal end). Measurements were made at four stages of flagellum elongation; (1) when the new flagellum tip was between the pocket and the nucleus, (2) when it was adjacent to the nucleus, (3) when it was anterior to the nucleus, and (4) when it was anterior to the mitochondrion at the anterior end of the cell (Fig. 5D, labeled 1–4, respectively). In the first of the four stages, close to the pocket and posterior to the nucleus (Fig. 5D, labeled 1) only up to 100 nm of the tip was embedded within the cell. This increased as the new flagellum elongated (statistically significant at *P*<0.001, *n* = 16 cells) until ∼500 nm of the flagellar tip was completely enclosed by the groove when the flagellum tip was close to the end of the cell (Fig. 5D, labeled 4).

## Discussion

Throughout growth of a new flagellum in the procyclic form of the parasite, the distal tip is tethered to the old flagellum through a mobile transmembrane junction – the flagella connector. This junction connects two flagella that are external to the cell body through filaments that extend from the growing microtubules of the axoneme in the new flagellum to the sides of three doublet microtubules in the old flagellum. In this paper, we describe an entirely different mechanism of distal tip attachment in the bloodstream form of the parasite. This distal tip is located in a discrete indentation of the cell body plasma membrane that we have named the groove. During growth of the new flagellum in the bloodstream form, the distal tip remains within the groove, representing an entirely novel mobile indented and differentiated area of the plasma membrane. Unlike the flagella connector of the procyclic form, in which the two flagella membranes are connected, this is a junction between the flagellum and the cell body plasma membrane, thereby connecting two plasma membranes of distinctly different compositions (Maric et al., 2010).

The cytoplasmic face of the plasma membrane of the groove is characterized by a network of electron densities with filaments radiating out from each density that interconnect with each other and with the cell body plasma membrane. Similar structures are observed in other cytoskeleton–membrane networks, for example, the erythrocyte membrane cytoskeleton. Erythrocytes have a spectrin-based cytoskeleton consisting of a meshwork of spectrin filaments converging on ankyrin/actin/Band 3-containing nodes linking the meshwork with the plasma membrane (Bennett, 1985; Li et al., 2007; Nans et al., 2011), which is ultrastuctrurally similar to the network observed in the groove. This specialized membrane cytoskeleton provides the erythrocyte with the ability to undergo large reversible deformations of the plasma membrane while maintaining surface area. These proteins are not present in the trypanosome. However, a structural arrangement of analogous proteins might play a role in providing a dynamic cytoplasmic-facing complex that allows connections to the microtubule cytoskeleton to be broken and remade as the flagellum tip grows forwards. In addition, this region would provide a discrete environment that defines a structural domain of the cell body membrane able to provide an attachment point for the new flagellum tip. A mesh-like inner filament cytoskeleton could also support the opening of the microtubule corset and restrict the degree of tip penetration into the cytoplasm. A filament–node meshwork makes intuitive sense in that it has the ability to be dynamic to accommodate movement of the new flagellum tip while still defining a definitive junctional complex.

Our tomography data revealed that the FAZ terminated at the point where the membrane invagination of the groove occurred, yet the flagellar and cell body plasma membranes were closely apposed throughout the entire area of the groove. Filament connections were observed on both the flagellar and cell body plasma membranes in the groove, suggesting a role in flagellum attachment within the groove. The distinctive pattern of labeling by the anti-FAZ antibody DOT1 at the area close to the distal tip of the growing new flagellum of the bloodstream form strongly suggests that there is some shared biochemistry with the FAZ filament. However, this labeling pattern was not consistent with DOT1 being localized throughout the entire area of the groove, so the filament connections within the plasma membrane might not be merely an elaboration of the linear FAZ filament. In addition, filaments on the flagellum side of the attachment region at the distal tip of the new flagellum were also unlike those previously defined for the flagellar side of the FAZ connecting to the PFR (Vickerman, 1969b). The PFR was not present in the distal tip of the growing new flagellum in the bloodstream form, and the filaments that were present connected with ultrastructurally indistinct material at the distal tip of the assembling axoneme.

**Fig. 6.**
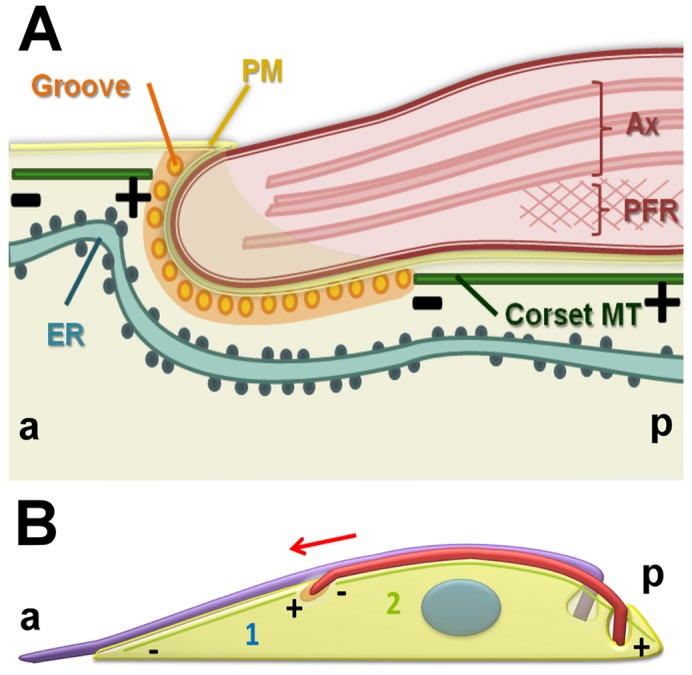
**A diagrammatic representation summarizing our interpretation of groove ultrastructure and subpellicular microtubule remodeling.** (A) Longitudinal view showing the distal tip of the new flagellum embedded in a groove. Orientation is as indicated (a, anterior and p, posterior). Microtubules terminate with their plus ends anterior to the groove (+). The plasma membrane (PM), axoneme (Ax) and paraflagellar rod (PFR) are shown. (B) Diagram representing a hypothetical remodeling of microtubules around the groove through disassembly (deploymerization or severing) of the plus ends of microtubules located anterior to the tip of the new flagellum (1). The new flagellum is elongating in the direction of the red arrow. Assembly and growth of microtubules posterior to the groove might occur at the minus end immediately posterior to the groove or at the plus end, which is at the posterior end of the cell (2).

Unlike the spectrin-based cytoskeleton that covers the entire cytoplasmic face of the erythrocyte cell, the groove is a mobile invagination with an entirely different ultrastructure to the subpellicular cytoskeleton that surrounds it. The subpellicular microtubules are cross-linked to one another and to the plasma membrane (Gull, 1999; Hemphill et al., 1991; Vickerman, 1985). Progression of the groove along the cell body as the new flagellum tip progresses clearly requires significant re-modeling of this microtubule array, and Fig. 6 outlines a model for this. Subpellicular microtubules were found to terminate anterior to the groove. The arrangement of microtubule polarity within the planar array of the subpellicular microtubules would predict these to be plus ends (Fig. 6A,B, labeled 1 in B) (Robinson et al., 1995); thus there must be a coordinated disassembly of individual microtubules and associated cross-linking proteins to accommodate the advancing groove. Microtubules were also observed to terminate immediately posterior to the groove (Fig. 6A,B), demonstrating a second area of significant remodeling. It is unlikely that these microtubules have an opposite polarity to the rest of the microtubules in the subpellicular array. There are at least two alternative hypotheses for remodeling of microtubules behind the path of the groove. It is possible that polymerization occurs at the minus ends of these microtubules at the same rate as growth of the new flagellum in order to ‘fill in’ behind the advancing groove (Fig. 6B, labeled 2). Alternatively, microtubule growth could occur at the plus ends, located at or close to the posterior end of the cell (Fig. 6B, labeled 2) and the microtubules could then slide forward within the array.

In procyclic cells, the flagella connector provides a cytotactic mechanism for influencing inheritance of cellular pattern and form as the cell divides (Beisson, 2008; Beisson and Sonneborn, 1965; Moreira-Leite et al., 2001; Sonneborn, 1964). The discovery of the groove in the bloodstream form now defines a structure that is likely to have a similar cytotactic role, yet deriving guidance from the underlying helically arranged subpellicular array or FAZ. It is intriguing that two trypomastigote life cycle forms have evolved different mechanisms for maintaining the growing new flagellum close to the cell while undergoing elongation. The distinct invagination of the groove is likely to have evolved with the parasitic lifestyle of the bloodstream trypomastigote. The *T. brucei* trypomastigote appears to have a fundamental requirement for flagellum tip growth being guided by connection to an external template. Presumably this is an influence of the complexity of cellular morphogenesis involving a long attached flagellum. The flagella connector mechanism used in the procyclic form trypomastigote has some possible disadvantages when seen in the context of a hostile immune response in the mammalian bloodstream.

The flagella connector must link both flagellar membranes together and we have described an interstitial layer that is external to these membranes (Briggs et al., 2004). Molecular models suggest that procyclin is an extended molecule and projects further from the membrane than the VSG molecules of the bloodstream form (Schwede et al., 2011). Hence, there is a possibility that, in the bloodstream form, a discrete flagella connector structure might be exposed to antibody recognition. Furthermore, the flagellum-to-flagellum connection is resolved by the splitting of the flagella connector, leaving a component exposed on each face (Davidge et al., 2006). Such an exposed pad of material in the bloodstream form would surely provide antigenic recognition opportunities for the immune system. Finally, although some invariant surface proteins of bloodstream forms can be recognized by the immune system (the ISGs for instance) they are likely to be present at such low density in the VSG layer that antibody cross-linking is unlikely. However, antibody recognition of a discrete flagella connector is more likely to lead to antibody cross-linking because of the densely packed externally exposed proteins. Designing a privileged enclosed site (analogous to the flagellar pocket for receptors) could have been an advantageous adaptation to bloodstream parasitism.

In conclusion, we have discovered a novel mobile junction within the cell body at the distal tip of the new flagellum of the bloodstream form. The differences in arrangements at the tip of the growing new flagellum in the procyclic and bloodstream forms of *T. brucei* trypomastigotes could explain many differences in division and cytokinesis RNAi mutant phenotypes between the two forms of this parasite. This discovery will enable further work to understand the biochemistry of this novel mobile junction and the nature of the massive remodeling of the membrane cytoskeleton connections that occur during morphogenesis of the bloodstream trypomastigote.

## Materials and Methods

### *T. brucei* cultivation and preparation of cells for electron microscopy

427WT bloodstream *T. brucei cells* were grown in an asynchronous culture in HM19 (Invitrogen, Paisley, Renfrewshire, UK) medium supplemented with 15% fetal calf serum (FCS) (Sigma, Gillingham, Dorset, UK) (37°C under 5% CO_2_) and were harvested at mid-log phase. Cells were fixed in suspension using 2.5% glutaraldehyde (TAAB, Aldermaston, Berkshire, UK) in the culture medium. After 3–5 minutes of fixation, cell pellets were re-suspended in a primary fixative containing 2.5% glutaraldehyde, 2% paraformaldehyde (Agar Scientific, Stansted, Essex, UK) and 0.1% tannic acid (TAAB) in 0.1 M phosphate buffer (Sigma) (pH 7.0) and centrifuged for 3 minutes. A fresh primary fixative solution was applied to the cell pellet and the cells were fixed for 2 hours at room temperature. Pellets were washed with 0.1 M phosphate buffer (pH 7.0) and post-fixed in 1% osmium tetroxide (Agar Scientific) in 0.1 M phosphate buffer (pH 7.0) for 1 hour at room temperature. The samples were rinsed and stained *en bloc* for 40 minutes in 2% uranyl acetate (TAAB), dehydrated in an ascending acetone series (Fisher Scientific, Loughborough, Leicestershire, UK) and embedded in Agar 100 resin (Agar Scientific).

### Electron tomography

Serial 200-nm sections of the embedded samples were collected onto formvar-coated slot grids and triple stained with 2% aqueous uranyl acetate, Reynolds leads citrate and uranyl acetate for a second time. 10-nm-diameter gold fiducials (BBI, Cardiff, UK) were placed on either side of the sections. Electron tomography was performed using a Hitachi H-7650 transmission electron microscope (Hitachi, Maidenhead, Berkshire, UK), operated at 120 kV. The images were recorded at zero defocus using an AMT 2k×2k CCD camera (Advanced Microscopy Technologies, Bury St. Edmunds, Suffolk, UK) at an original magnification of 15,000×. Single axis and dual axis image series were collected from −60° to +60° tilt along alpha and beta tilt axes at 2° increments. For serial section tomography, single and dual tilt data was collected from the same cell across 2–5 adjacent sections. Images in each tomography tilt series were aligned and combined to generate 3D tomograms using eTomo (IMOD, Boulder, CO, USA) (Mastronarde, 1997). The data were aligned using standard gold-bead tracking using 10–20 fiducials, and tomogram generation used the weighted back-projection reconstruction technique. For dual-tilt data sets, the two tomograms generated for each axis were aligned using the fiducials and combined into a single tomogram. Serial tomograms were then combined using the ‘New Join’ function, and microtubules were used to refine the final alignment and create a single final tomogram. To reduce image noise, the tomograms were first filtered using the median function of the clip IMOD program. Segmentation and image processing were conducted using Amira 5.4.2 (Visualisation Sciences Group, Düsseldorf, Germany).

### SBF-SEM

SBF-SEM is a relatively new technique in electron microscopy, using a diamond knife mounted within a scanning electron microscope to serially section a resin block. The cut block face is imaged between each slice using a backscattered electron detector (Denk and Horstmann, 2004). Resin-embedded samples were trimmed and placed into a Quanta 250 FEG (FEI, Eindhoven, Netherlands) with a fitted Gatan 3view system (Gatan, Abingdon, Oxfordshire, UK). Serial images of the block face were recorded at an accelerating voltage of 5 kV, a spot size of 2.5 and pressure of 0.5 Torr. The dwell time for each micrograph was 5 µs. Pixel size was 6–6.2 nm and slice thickness was 100 nm. Images were recorded using Digital Micrograph (Gatan, Abingdon, Oxfordshire, UK). Images were combined into an mrc file format using IMOD. Individual whole cells were selected and boxed out using the trimvol function of IMOD.

### Data processing

Distinct characteristics of the data were selected for segmentation, the density of stain and structural morphology, using a combination of automatic (thresholding and interpolation) and manual (‘brush’) tools. The movies shown in the supplementary material were generated using the demo-maker and movie-maker functions on Amira and the measurement tool used to take measurements. Movies of tilt series and tomograms were generated using 3Dmod (IMOD, Boulder, CO). VideoMach (Version 5.9.0, Gromada) was used to combine images into movies.

Measurements for the length of flagellar tip enveloped by the groove were conducted on SBF-SEM data using the 3D measuring tool on Amira. Measurements were taken from the centre of the axoneme to the centre of the distal tip from the point where the entire flagellum was enclosed by the cell. The measurements were divided up into stages of flagellar elongation as per the diagram shown in Fig. 2C on the basis of the surrounding ultrastructure; when the new flagellum tip was between the flagellar pocket and the nucleus (1), adjacent to the nucleus (2), anterior to the nucleus (3) and anterior to the mitochondrion (4). A total of 4–5 flagellar were measured for each stage, and a single-factor ANOVA statistical test was conducted. The *P*-value for ‘between group’ variation was <0.0001.

### Immunofluorescence microscopy

WT427 cultured bloodstream form and procyclic form cells from mid-log cultures were used. Cells were extracted with 1% NP-40 in 100 mM PIPES (Melford, Ipswich, Suffolk, UK) pH 6.9, 2 mM EGTA (Fisher Scientific), 1 mM MgSO_4_ (Fisher Scientific) and 0.1 mM EDTA (Fisher Scientific) (PEME) for ∼1 minute. The cytoskeletons were fixed in methanol (Fisher Scientific) at −20°C for 30 minutes, blocked with 1% BSA (Sigma) and labeled with with L3B2 (FAZ1, 1∶2 dilution) or DOT1 (applied neat) for 40 minutes at room temperature. The FITC-conjugated anti-mouse-Ig (DAKO, Ely, Cambridgeshire, UK) (1∶50 dilution) secondary antibody was applied for 40 minutes. Cells were embedded in Vectashield (Vector Laboratories, Peterborough, Cambridgeshire, UK) with 4,6-diamidino-2-phenylindole (DAPI) (Vector Laboratories) to visualize nuclear and mitochondrial DNA. Images were captured on a DM5500 B epifluorescence microscope (Leica Microsystems, Milton Keynes, Buckinghamshire, UK) with an Orca cooled CCD camera (Hamamatsu Photonics, Welwyn Garden City, Hertfordshire, UK).

## Supplementary Material

Supplementary Material
