# Supplementary material for: A cell-body groove housing the new flagellum tip suggests an adaptation of cellular morphogenesis for parasitism in the bloodstream form of Trypanosoma brucei
Source: J Cell Sci. 2013 Dec 15;126(24):5748–57. doi: 10.1242/jcs.139139 (PMC3860315; doi:10.1242/jcs.139139)
Supplement: Supplementary Material [file supp_126_24_5748__index.html]

A cell-body groove housing the new flagellum tip suggests an adaptation of cellular morphogenesis for parasitism in the bloodstream form of Trypanosoma brucei — Supplementary Material 

# A cell-body groove housing the new flagellum tip suggests an adaptation of cellular morphogenesis for parasitism in the bloodstream form of *Trypanosoma brucei*

## JCS139139 Supplementary Material

**Files in this Data Supplement:**

- **Supplementary Material PDF**
