# Supplementary material for: A cell-body groove housing the new flagellum tip suggests an adaptation of cellular morphogenesis for parasitism in the bloodstream form of Trypanosoma brucei
Source: J Cell Sci. 2013 Dec 15;126(24):5748–57. doi: 10.1242/jcs.139139 (PMC3860315; doi:10.1242/jcs.139139)
Supplement: Supplementary Material [file supp_126.24.5748_JCS139139.pdf]

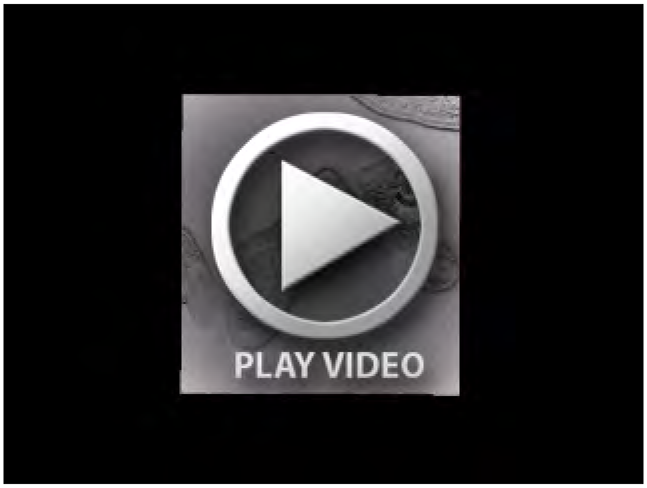

Movie 1. Combined serial section tomogram and surface rendering of segmented data (also shown in Fig. 2A). The groove occurs in a gap in the subpellicular microtubule corset. Microtubules terminate in advance of the groove, maintaining regular spacing of the subpellicular corset. Colour legend: subpellicular microtubules (green), MtQ (light green), punctuate electron densities of the groove (orange), FAZ filament (blue), old flagellum membrane (purple), new flagellum membrane (red), axoneme microtubules (white), cell body plasma membrane (transparent yellow).

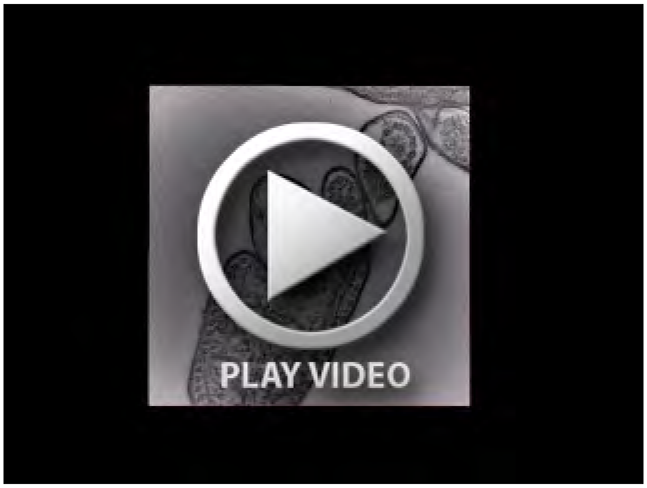

Movie 2. Combined serial section dual axis tomogram and surface rendering of segmented data (also shown in Fig. 2B). The movie shows the termination of subpellicular microtubules and new FAZ posterior to the groove.

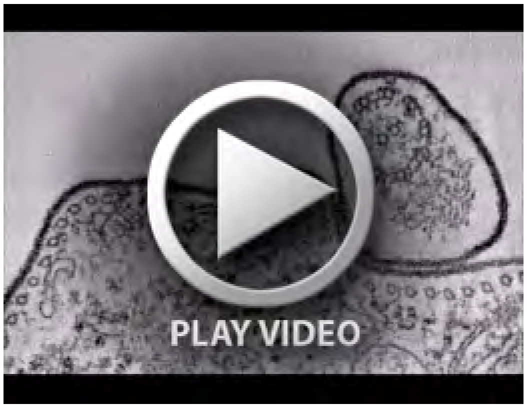

Movie 3. Sequential slices of a joined serial section dual-axis tomogram (also shown in Fig. 3A-K) illustrating the staggered assembly of microtubule doublets of the 9+2 axoneme.

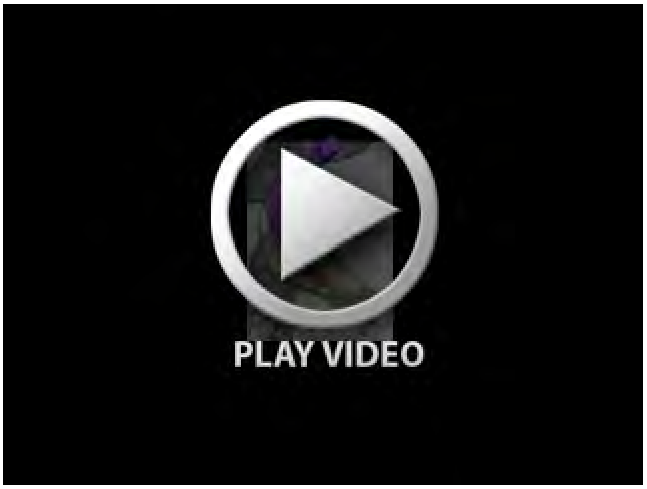

Movie 4. SBF-SEM whole cell data shown in Fig. 2A. Segmentation and SBF-SEM data are merged. The movie shows a new flagellum emerging from the flagellar pocket with the distal tip embedded in a groove.

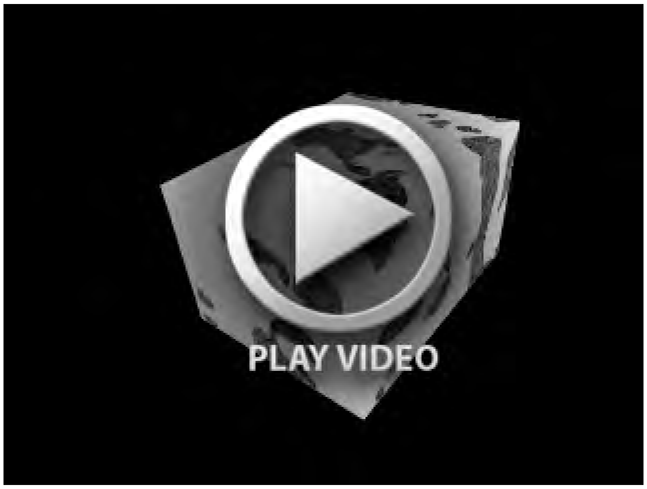

Movie 5. SBF-SEM whole cell data also shown in Fig. 5C. Segmentation and SBF-SEM data are merged. The movie shows the surface of a whole cell and the invagination of the distal tip of the new flagellum (red) into a groove.
